# [Retracted] MACC‑1 antibody target therapy suppresses growth and migration of non‑small cell lung cancer

**DOI:** 10.3892/mmr.2023.13130

**Published:** 2023-11-20

**Authors:** Woda Shi, Jianxiang Song, Wencai Wang, Yajun Zhang, Shiying Zheng

Mol Med Rep 16: 7329–7336, 2017; DOI: 10.3892/mmr.2017.7517

Following the publication of this paper, it was drawn to the Editor's attention by a concerned reader that the control data in Fig. 2B and C on p. 7332, showing immunofluorescence and migration assay experiments respectively, were strikingly similar to data appearing in different form in another article which was written by different authors at different research institutes [Tian L, Shen D, Li X, Shan X, Wang X, Yan Q and Liu J: Ginsenoside Rg3 inhibits epithelial-mesenchymal transition (EMT) and invasion of lung cancer by down-regulating FUT4. Oncotarget 7: 1619–1632, 2016].

Owing to the fact that the contentious data in the above article had already been published prior to its submission to *Molecular Medicine Reports*, the Editor has decided that this paper should be retracted from the Journal. The authors were asked for an explanation to account for these concerns, but the Editorial Office did not receive a reply. The Editor apologizes to the readership for any inconvenience caused.

